# Biodiversity of Local *Vitis vinifera* L. Germplasm: A Powerful Tool Toward Adaptation to Global Warming and Desired Grape Composition

**DOI:** 10.3389/fpls.2020.00608

**Published:** 2020-05-14

**Authors:** Tommaso Frioni, Giovanni Bertoloni, Cecilia Squeri, Alessandra Garavani, Lily Ronney, Stefano Poni, Matteo Gatti

**Affiliations:** Dipartimento di Scienze delle Produzioni Vegetali Sostenibili, Università Cattolica del Sacro Cuore, Piacenza, Italy

**Keywords:** autochthonous cultivars, minor varietals, viticulture, climate change, titratable acidity, malic acid

## Abstract

Global warming is endangering maintenance of optimal grape composition in white varietals aimed at sparkling wine making due to difficulties to maintain adequate acidity and fresh aromas. These troubles are being faced by the main white varietal of the Colli Piacentini district, named Ortrugo. Its vegetative and reproductive behavior was compared over 3 years with that of other minor autochthonous white varietals. Criteria set for adequate grape composition under sparkling vinification (total soluble solids at 20–21°Brix) and titratable acidity (TA) ≥ 6.5 g/L combined with Principal Component Analysis (PCA) on the measured variables allowed a thinnning down of the initial group of 17 to 7 varietals including Ortrugo, Bucalò, Barbesino, Lecco, Melara, Santa Maria and Molinelli. PCA isolated Ortrugo’s behavior for inadequacy to maintain sufficient TA at harvest mostly due to extremely low malic acid concentration. However, time trend analyses of accumulation and degradation patterns of tartaric and malic acids disclosed that, in Ortrugo, the most limiting factors were more intense post-veraison tartaric acid dilution and a lower malic acid pool at veraison as compared to any other varietal. Conversely, Molinelli and Barbesino proved to be ideal material for sparkling wine purposes, as they associated to desirable agronomic features a strong ability to retain high TA with a well-balanced tartrate-to-malate ratio. Our study emphasizes that often neglected or superficially evaluated germplasm genetic resources might hide strong potential for adapting to challenges imposed by climate change in that representing an excellent tool for adaptation strategies.

## Introduction

Effects of climate change on viticulture have been the object of a massive flux of scientific literature since the mid-80s’ (Mozell and Thach, 2014; Palliotti et al., 2014; Schultz, 2016; Van Leeuwen et al., 2019; De Ollas et al., 2019). Global warming is also, albeit slowly, re-designing the geographical distribution of cultivars (i.e. former cool areas can nowadays accommodate medium or late ripening varieties) whereas warm areas are often facing excessive light and heating availability as compared to the need of traditionally grown varieties (Jones and Webb, 2010; Schultz, 2016; Van Leeuwen et al., 2019). Moreover, the issue of “meteorological drought” is rising all across Europe (Spinoni et al., 2016) and territories that have been dry farmed for ages are now facing the uncomfortable issue of having to provide supplemental water with irrigation (Van Leeuwen et al., 2019). Other dramatic impacts of climate change in warm viticulture areas include: (i) increased advancement and compression of all phenological stages; according to Sadras and Petrie (2012) number of days (d) elapsing between average harvest dates for Chardonnay and Cabernet Sauvignon cultivars has decreased from 21 days at the beginning of the 90 s to only 9 days; (ii) increasing frequency of extreme events (e.g. mild winters, severe summer drought, hot spells, unexpected unseasonal flooding etc.) that might have serious effects on viticulture profitability. A suitable example is recurrent frost damage fostered by very early bud push and, afterward, a longer time window during which a damaging frost event might still occur; (iii) within a global change scenario, ideal grape composition for some specific wines is definitely more difficult to achieve (Poni et al., 2018).

In the case of white varieties, and especially to those grown to produce sparkling or spumante wines, the challenge thrown by climate change is of utmost complexity. If the wine target is a sparkling, a desirable grape composition at harvest is often as follows: a total soluble concentration (TSS) between 20 and 21°Brix, a titratable acidity (TA) ≥ 6.5–7.0 g/L, a pH ≤ 3.2 and, hopefully, healthy and turgid berries. Among such desired characteristics, increased heat summations – including also a marked increase of night temperatures – primarily endanger the maintenance of adequate acidity. It is well established (Ruffner, 1982; Ford, 2012) that, while tartaric acid concentration is essentially unaffected by temperature, the rapid loss in malic acid starting at the onset of berry softening is mainly temperature driven (Ford, 2012).

If the goal is to maintain adequate total acidity at harvest with a good balance between the two main organic acids of the grape berry, then genetic variation in organic acid metabolism should be investigated and eventually exploited. It has been known for some time that species within the genus *Vitis* and individual varieties of the cultivated grapevine *V. vinifera* show ample variation in the natural acidity of berries (Duchéne, 2016; Poni et al., 2018; Famiani et al., 2016, 2018). Analysis of the acid composition in developing and ripe berries of 26 species of *Vitis* and 50 wine grape varieties of *V. vinifera* showed that, for a so called “early” sample, malic concentration ranged from a maximum of 0.85 g/100 mL in Pinot St George to the lowest 0.26 g/100 mL recorded in White Riesling. At late sampling, the majority of the varieties showed malate levels below 0.15 g/100 mL (Kliewer et al., 1967). Most importantly, though, some varieties showed greater than 50% loss of malate between early and late sampling, whereas others (e.g. White and Gray Riesling) retained malate at comparable levels across the sampling period.

Even if most of Italian and southern Europe viticulture is based on local varieties identifying the final products, the re-arrangement of cultivars and genotypes adopted within a wine region is something currently occurring especially as a consequence of climate change pressures (Palliotti et al., 2014; Mosedale et al., 2016). However, even if in literature some works report the evaluation of local biodiversity and the re-introduction of autochthonous minor cultivars as new tools to improve the competitiveness of wine districts (Mannini, 2003; Storchi et al., 2007; Cruz-Castillo et al., 2009; Iorizzo et al., 2014; Biasi and Brunori, 2015; Urrestarazu et al., 2015; Gutiérrez-Gamboa et al., 2020), none of them has taken into account white varietals and the importance of maintaining acidity in grapes in relation to high seasonal temperatures favoring abrupt organic acids depletion.

The local (autochthonous) white cultivar Ortrugo is currently grown over about 650 hectares in the Colli Piacentini wine district, in Northern Italy. Ortrugo has its own appellation and it is recognized as a high quality sparkling and spumante wine that is currently highly requested both nationwide and in export markets. Unfortunately, Ortrugo is extremely sensitive to post-veraison malate degradation and even in the presence of cultural practices aiming to screen cluster from direct radiation in summer (Gatti et al., 2015, 2019) in a typically warm season it is quite normal that TA in Ortrugo drops to unacceptable levels (e.g. ≤6.0 g/L with malic acid often below 0.5 g/L). Within the same region where Ortrugo is grown, several autochthonous, yet minor, white varietals have been isolated and today they are maintained in a private vineyard collection (Fregoni et al., 2002) officially recognized by the Emilia Romagna Region (including Begano Bianco, Bervedino, Marsanne, Melara, Ortrugo and Santa Maria, officially registered in the National Catalog of Grapevine Varieties RNVV, Barbesino, Bucalò, Calöra, Bianchetta di Diolo, Bianchetta di Bacedasco, Colombina, Molinelli, Lecco, Lisöra, Stciucaera Bianca currently not listed in the RNVV). “Minor” in the present case defines a varietal that, for a number of reasons (e.g. specialization in viticulture focusing on the most productive biotypes with progressive erosion of the local biodiversity, adverse climatic regimes leading to poor ripening of late varietals and higher disease pressure, as well as conservative policy making in terms of modifications of current appellation regulations), have been progressively neglected by growers, resulting in a drastic reduction of planted surfaces that is also conducive to the risk of total loss of propagation material.

Hypothesis pursued in this study is that biodiversity hidden within a population of local varieties can aid at solving the scarce performances of traditionally selected grapevine cultivars under the current climate change scenario. In this specific case, cv. Ortrugo, almost flattening acidity at harvest under warming trends pressures, was tested versus 16 other minor white varietals for vegetative parameters, yield and fruit composition at harvest; “developmental sampling” of berries across multiple time points in the growing seasons was undertaken to provide comparison of acid levels vs other parameters of technological maturity and to explore the physiological and metabolic reasons hidden behind differential ripening patterns.

## Materials and Methods

### Experimental Site and Treatment Layout

The study was carried out for 3 years (2017–2019) in a vineyard germplasm collection planted in 2003 at the Mossi Estate (Albareto, Ziano Piacentino, Italy, 44° 97′ 93″ N 09 40′ 99″ E, 270 m asl) where, along with Ortrugo, 16 more white minor varietals (Supplementary Figure S1) are reunited. All varietals, grafted on K5BB rootstock, are planted at 2.2 × 2 m spacing (between row and within row distance, respectively), with coupled vines in the row for a resulting density of 4545 plants/hectare. Rows are directed along maximum soil slope (about 12%) assuming a SE-NW orientation and vines are trained to a unilateral Guyot with about 10 nodes on the primary horizontal cane and two more on a spur left for annual cane renewal. According to the relative abundance of the propagation material, each varietal is present in at least one or two adjacent rows. The experiment was conducted on 30 vines per genotype (510 vines total) randomly chosen within the available plots. Each year, thinning was applied between BBCH 14–15 to maintain one primary shoot per node and four test vines per variety were randomly chosen along the row(s) in 2017 and then maintained also for the two following seasons. These selected vines were used for detailed assessment of vegetative growth, yield components and grape composition at harvest, whereas the others were used for veraison-to-harvest berry samplings. The vineyard is typically non-irrigated, whereas fertilization and disease and pest management were uniform across the whole vineyard surface and conducted according to local sustainable practices. The minimum, mean, and maximum daily air temperature (°C) and daily rainfall (mm) from 1 January (DOY 1) to 31 December (DOY 365) were recorded in each season by a nearby weather station.

### Vegetative Growth and Yield Components

Upon completion of leaf fall (end of November) all test vines were pruned and the removed 1-year old pruning weight immediately recorded in the field with a portable digital scale.

Each season, in late spring (end of May – beginning of June), number of inflorescences borne on each shoot was recorded according to position of the shoot on the horizontal cane. Total vine fruitfulness was then calculated as a ratio of total inflorescences on total shoots.

At harvest, test vines were individually picked and total cluster number per vine counted. Concurrently, three representative clusters per vine, usually inserted on basal, median and apical cane portions, were taken to the laboratory for further subsampling. Fruits were individually weighted and the main rachis length measured in order to calculate the compactness index expressed as cluster mass-to-rachis length ratio (Tello and Ibanez, 2014). From each of the three clusters, one 50 berry sub-sample was taken by careful cutting each berry at the pedicel with small sharp scissors and then crushed. The obtained must was then used for technological maturity determinations as described in the next paragraph. In each year, the yield to total pruning weight ratio (kg/kg), otherwise known as Ravaz index (Ravaz, 1911) was calculated on a single vine basis.

### Grape Composition

Each year, from veraison (TSS ∼4.5 to 5 Brix) until harvest, three 50-berry samples were taken weekly from extra vines of each varietal. Test vines were excluded to not alter natural dynamic of grape ripening due to progressive reduction of the pending yield. During sampling, it was assured that the removed berries were taken from clusters located on both sides of the row and, within each cluster, the top, median, and bottom portions were also represented. Sampled berries were brought to the laboratory, weighed, and crushed to obtain a juice. Musts were analyzed immediately for TSS using a temperature-compensated desk refractometer, whereas pH and TA were measured by titration with 0.1 N NaOH to a pH 8.2 end point and expressed as g/L of tartaric acid equivalents.

In each season, all varietals were picked at similar ripening aiming at a final TSS of about 20–21°Brix and a TA ≥ 6.5 g/L. Resulting harvest dates were 22 August 2017, 30 August 2018 and 5 September 2019. TSS, TA, and pH were determined on the remaining berries of each of the three sampled clusters according to the standard methods described above.

### HPLC Analysis

To assess tartaric and malic acid concentrations in all samples taken seasonally and at harvest, an aliquot of the must was diluted four times, then filtered through a 0.22 μm polypropylene syringe for high-performance liquid chromatography (HPLC) analysis and transferred to auto-sampler vials. All solvents were of HPLC grade. Water Milli-Q quality, acetonitrile, and methanol were obtained from VWR. L-(+)-tartaric acid and L-(-)- malic acid standards were purchased from Sigma-Aldrich. The chromatographic method was developed using an Agilent 1260 Infinity Quaternary LC (Agilent Technology) consisting of a G1311B/C quaternary pump with an inline degassing unit, G1329B autosampler, G1330B thermostat, G1316B thermostated column compartment, and a G4212B diode array detector (DAD) fitted with a 10 mm path, 1 μL volume Max-Light cartridge flow cell. The instrument was controlled using the Agilent Chemstation software version A.01.05. The organic acids’ analysis used an Allure Organic Acid Column, 300 × 4.6 mm, 5 μm (Restek). Separation was performed in isocratic conditions using water, pH-adjusted to 2.5 using ortho-phosphoric acid, at a flow rate of 0.8 mL/min. The column temperature was maintained at 30 ± 0.1°C, and 15 μL of sample was injected. The elution was monitored at 200 to 700 nm and detected by UV-vis absorption with DAD at 210 nm. Organic acids were identified using authentic standards, and quantification was based on peak areas and performed by external calibration with standards.

### Statistical Analysis

Vine performance data were subjected to a two-way analysis of variance (ANOVA) using the SigmaStat software package (Systat Software, Inc.). Homogeneity of error variances for data taken on the same individuals over different years was assessed with Bartlett’s test. The year was considered as a random variable, and the error term for the treatment factor was the year × treatment interaction mean square. Since variances were in all cases homogeneous, the year × treatment effects were tested using the pooled error mean square as an error term (Gomez and Gomez, 1984). Treatment comparison was performed using the Student-Neuman-Keuls test at *p* ≤ 0.05. Year × treatment interaction was partitioned only when the F test was significant.

Due to the high number of measured variables, a Principal Component Analysis (PCA) was also carried out twice using the XLSTAT statistical package (Addinsoft, New York, NY, United States). In the first run, observations were single vine data for the 7 selected varietals, whereas 11 variables were analyzed to include parameters representative of vine vigor, yield and grape composition. The second run was performed on the yearly data of the same varietals, additionally associating another set of seven variables representative of sugar and acid seasonal dynamics and climatic indices. In both cases, the chosen PCA was a Pearson correlation matrix, number of filter factors was set at 5 and the final data visualization was in the form of a distance bi-plot.

Repeated measures of the same parameters (berry mass, TSS, TSS/berry mass, TA, tartrate, malate) taken at different dates, throughout the season were analyzed with the Repeated Measure analysis of variance (ANOVA) routine embedded in the XLSTAT software package. The least squared (LS) mean method at *p* ≤ 0.05 was used for multiple comparisons within dates.

## Results

### Weather Trends and Indices

In 2017, total rainfall recorded from April to October summed up to 289 mm (Supplementary Figure S2 and Supplementary Table S1). Very limited rainfall occurred in summer months and, in July and August, 13 days registered T_max_ higher than 35°C. The Winkler Index (WI) calculated over a 10°C baseline was of 2143 Degree Days (DD) which lowered to 1984 DD if the calculating period was shortened at the end of September. The 2018 season registered a higher rainfall from April to October (433 mm) than 2017; yet summer temperatures were fairly high and WI resulted in 2200 DD (2014 from April to September). Year 2019 will be remembered for an unseasonal wet spring (about 300 mm fell in April and May out of 595 mm from April to October); at the same time June and July were very hot (peak T_max_ of 39.5°C recorded on DOY 178). WI summed up to 2020 DD, whereas WI calculation restricted from April to September yielded 1963 DD.

### Vine Growth and Yield Components

Shoot number per vine was very constant across years, whereas it ranged from 9.9 (Besgano Bianco) to 13.8 (Molinelli) among varietals with Ortrugo setting at an intermediate position (11.8 shoots/vine) (Table 1). For data pooled over varietals, shoot fruitfulness had an overall mild decrease when calculated for the basal cane nodes (first three count nods) vs. total nodes. However, among cultivars, Bervedino scored the highest fertility (1.6 inflorescences/shoot) and Lecco the lowest (0.5 inflorescences/shoot). In Melara only, restricting calculation of shoot fruitfulness to the first three nodes resulted in a much lower value (0.2 inflorescences/shoot) as compared to total cane value (0.7 inflorescences/shoot). Total pruning weight per vine for data pooled over varietals showed that 2017 was the weakest season with only 299 g; large variation in pruning weight per vine occurred among genotypes with an almost four-fold difference between minimum (149 g) and maximum (581 g) values recorded in Stciucaera Bianca and Besgano Bianco, respectively, while Ortrugo set at 308 g.

**TABLE 1:** Vegetative growth, yield components and Ravaz Index recorded over 3 years (2017–2019) in 17 *Vitis vinifera* L. varietals including the reference cultivar Ortrugo. Data were taken on four vines per varietal.

Varietals	Shoots/vine	Total pruning weight/vine (g)	Cane fruitfulness (clusters/shoot)	Basal cane fruitfulness (clusters/shoot)	Yield/vine (kg)	Clusters/vine	Cluster Weight (g)	Cluster compactness (g/cm)	Berry weight (g)	Ravaz Index (kg/kg)
Barbesino	11.8 ab	449 ab	1.3 bcd	1.0 abcde	2.05 bcde	13.2 bc	158 cdefg	13.13 ab	1.98 bcdef	4.77 cde
Bervedino	12.3 ab	233 bcd	1.6 a	1.6 a	3.47 a	19.5 a	181 cdef	22.87 a	1.98 bcdef	18.68 b
Besgano Bianco	9.9 b	581 a	0.9 defg	0.7 bcde	2.12 bcde	9.2 bcd	231 abc	15.64 ab	3.43 a	4.54 cde
Bianchetta di Bacedasco	11.5 ab	381 abc	0.8 efgh	0.6 cde	2.22 bcde	10.5 bcd	216 abcd	13.69 ab	2.21 bcd	7.22 cde
Bianchetta di Diolo	11.6 ab	373 abc	1.0 def	1.1 abcd	3.00 ab	11.3 bcd	282 a	22.57 a	3.36 a	10.48 c
Bucalò	12.7 ab	253 bcd	0.6 hi	0.7 bcde	1.04 ef	7.7 de	139 efg	12.45 b	1.80 defg	5.19 cde
Calora	11.1 b	457 ab	1.0 defg	0.8 bcde	2.57 abc	13.1 bc	208 bcde	12.11 b	2.37 b	6.25 cde
Colombina	10.4 b	452 ab	1.1 def	0.8 bcde	2.14 bcde	12.3 bcd	177 cdef	12.05 b	1.52 *g*	5.85 cde
Lecco	10.8 b	297 bcd	0.5 i	0.4 de	0.42 f	4.4 e	100 g	8.65 b	1.96 bcdef	1.72 e
Lisöra	11.1 b	188 cd	1.4 abc	1.4 ab	1.46 cde	13.4 b	117 fg	13.25 ab	2.00 bcdef	9.14 *cd*
Marsanne	12.3 ab	273 bcd	1.2 cde	1.3 abc	1.88 bcde	12.6 bcd	141 defg	9.67 b	1.72 efg	7.16 cde
Melara	12.7 ab	413 ab	0.7 fghi	0.2 e	1.24 def	8 cde	165 cdefg	11.93 b	2.14 bcde	3.66 de
Molinelli	13.8 a	398 abc	1.5 ab	1.6 a	1.83 bcde	17.3 a	113 fg	7.97 b	1.62 fg	6.43 cde
Ortrugo	11.8 ab	308 bcd	0.7 ghi	0.5 de	2.37 bcd	8.1 cde	270 ab	18.23 ab	1.86 cdefg	8.60 *cd*
Santa Maria	11.1 b	441 ab	1.0 defg	0.7 bcde	1.53 cde	9.5 bcd	165 cdefg	13.01 ab	2.23 bc	3.64 de
Stciucaera Bianca	12.1 ab	149 d	0.9 defg	0.7 bcde	2.09 bcde	11.7 bcd	174 cdefg	11.57 b	2.17 bcd	23.54 a
Verdea	10.4 b	532 a	1.0 def	0.7 bcde	2.36 abc	10.1 bcd	231 abc	13.41 ab	1.94 cdef	4.85 cde
**Year**										
2017	11.5 a	299 b	1.1 a	1.0 a	2.33 a	13 a	177 b	14.86 a	2.14 b	12.14 a
2018	12.1 a	415 a	0.9 c	0.7 b	1.88 b	9 c	216 a	13.24 a	2.26 a	5.70 a
2019	11.4 a	390 a	1.0 b	1.0 a	1.85 c	12 b	156 c	13.17 a	2.05 b	5.48 b
**Varietal**	***	***	***	***	***	***	***	***	***	***
**Year**	*	***	***	**	**	***	***	ns	**	***
**V** × **Y**	ns	ns	ns	ns	ns	ns	ns	ns	ns	ns

Within each column and factor (varietals and year) mean separation was performed using the Student-Newman-Keuls test. Probability levels are: ***P < 0.001; **P < 0.01; *P < 0.05. ns implies non-significance.

Lack of significant V × Y interactions for any of the yield components reported in Table 1 indicates that performance of different varietals was rather uniform across the three seasons. Yield per vine fell between 0.42 kg in Lecco and 3.47 kg in Bervedino and high variability among cultivars was recorded also for cluster weight (100–282 g span) and berry weight (1.52–3.43 g span). Absolute values for cluster compactness also showed high variability among cultivars, yet within varietals mean separation highlighted very few significant differences. Likewise, the Ravaz index showed quite high variability as it went from 1.7 kg/kg (Lecco) to 18.8 kg/kg (Bervedino).

### Grape Composition

**FIGURE 1:**
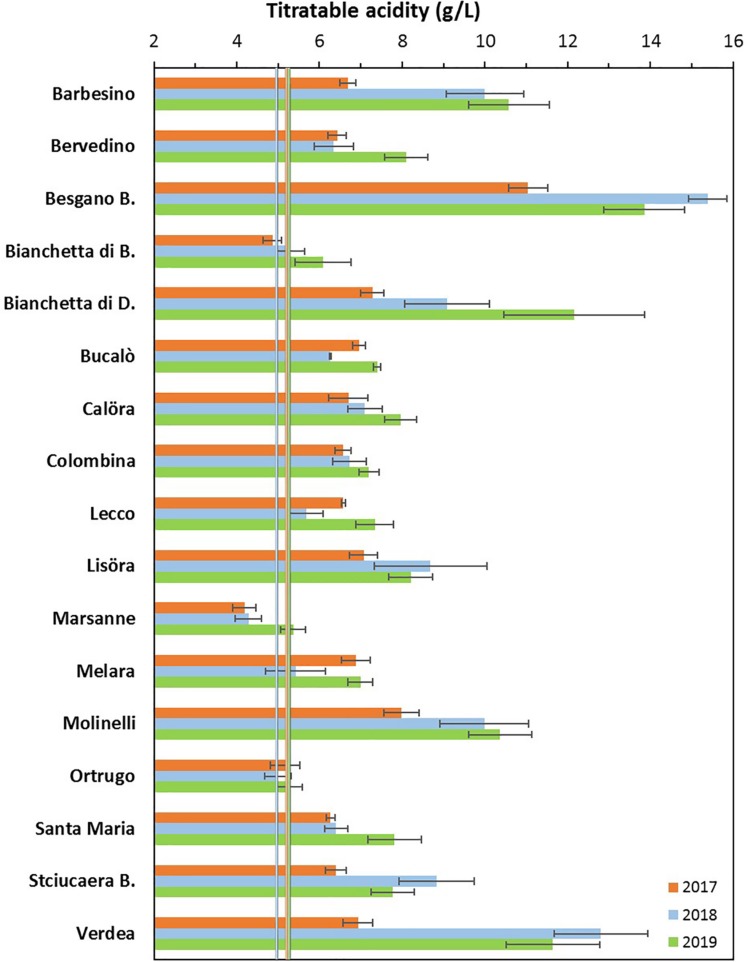
Interactive effects between genotype and year for must titratable acidity (g/L) at harvest in 17 *Vitis vinifera* L. varietals including the reference cultivar Ortrugo. Data are means ± and SE, *n* = 4.

**FIGURE 2:**
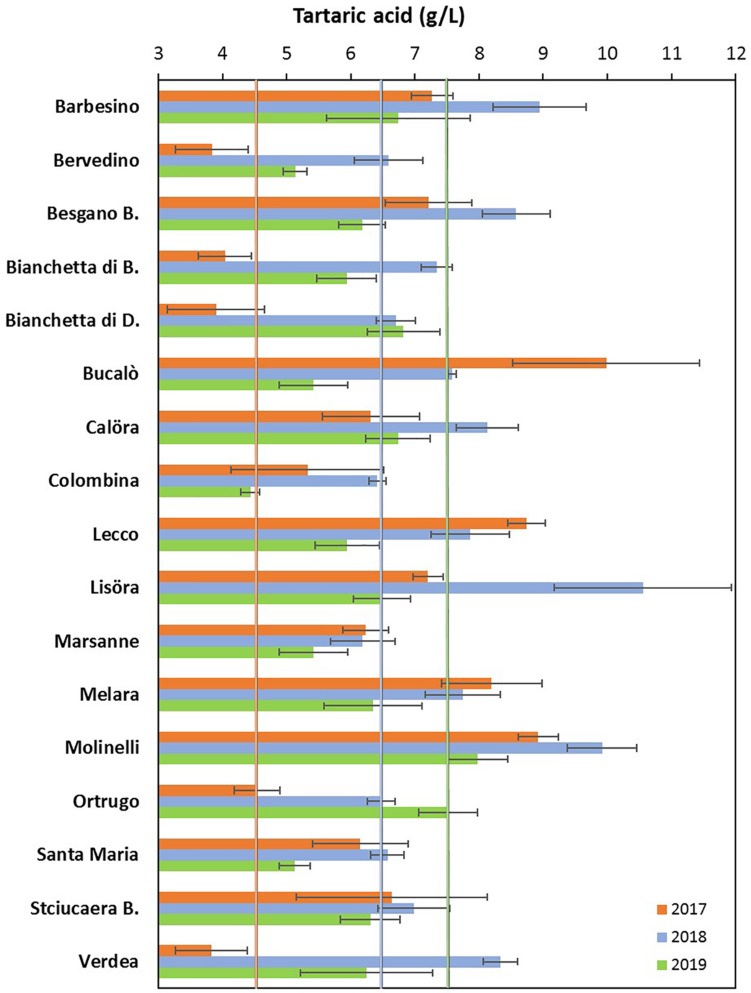
Interactive effects between genotype and year for must tartaric acid concentration (g/L) at harvest in 17 *Vitis vinifera* L. varietals including the reference cultivar Ortrugo. Data are means ± and SE, *n* = 4.

**FIGURE 3:**
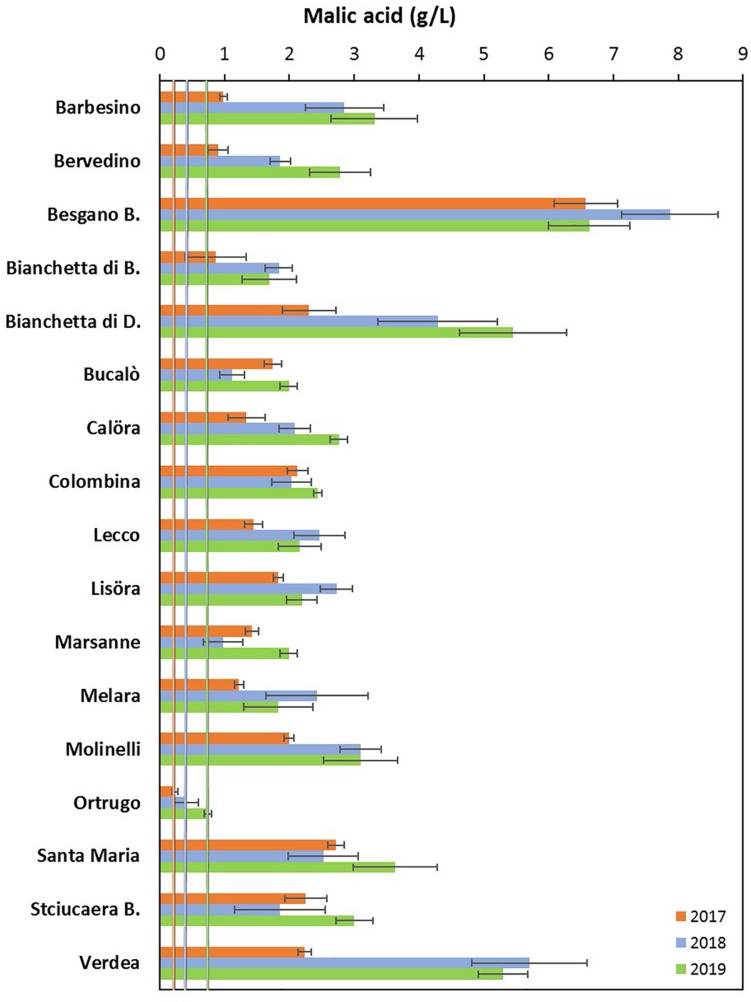
Interactive effects between genotype and year for must malic acid concentration (g/L) at harvest in 17 *Vitis vinifera* L. varietals including the reference cultivar Ortrugo. Data are means ± and SE, *n* = 4.

Total soluble concentration recorded at harvest have shown that five varietals, and namely Bucalò, Lecco, Marsanne, Melara and Molinelli reached higher berry sugar concentration than the reference Ortrugo (20.8°Brix) (Table 2). Must pH in almost all cases was lower than the 3.3 threshold. Within-varietals variability for acid components was very broad: TA ranged from the lowest 5.15 g/L scored by Ortrugo to the 13.4 g/L of Besgano Bianco; tartrate went from a minimum of 5.18 g/L in Bervedino to a maximum of 8.94 g/L in Molinelli and malate was the lowest in Ortrugo (0.46 g/L) and the highest in Besgano Bianco (7.0 g/L). Table 2 data analysis also shows that a significant V × Y interaction occurred for TA, tartrate, malate and tartrate/malate ratio (Figures 1–3). Partitioning of such interactions disclosed, for TA, that the majority of varietals was sensitive to seasonal effects leading to a lower TA retaining in the hot and dry 2017 as compared to the following two seasons (see for reference, main effects for year factor in Table 2). Some varietals, though, were less responsive to such climate-driven effect and their TA was relatively stable across years; among them Bucalò, Colombina, Lecco, Melara and Ortrugo (Figure 1). Tartaric acid concentration at harvest showed a somewhat different response to yearly effects among varietals; in fact, the pattern described above for TA was maintained only for Bervedino, Bianchetta di Bacedasco (Bianchetta di B.), Bianchetta di Diolo (Bianchetta di D.), Ortrugo and Verdea. In all remaining varietals, tartrate in 2017 was either similar or even higher than the concentration determined at harvest in 2018 and 2019 (Figure 2). Malate concentration at harvest confirmed high variability in terms of single varietal sensitivity to the climatic patterns; the hot and dry 2017 did not necessarily result in lowest malic acid concentration at harvest (cases of Besgano Bianco, Bucalò, Colombina, Marsanne, Santa Maria, Stciucaera Bianca) (Figure 3). Ortrugo had a very distinctive behavior, since in no years the malic acid concentration reached the threshold of 1 g/L. Variation of the calculated tartrate/malate ratio was an obvious consequence of relative changes of the main acids and Ortrugo, with a ratio of 18.3, outclassed all remaining varietals whose ratios ranged between 1.1 and 5.3 (not shown).

**TABLE 2:** Parameters of technological maturity recorded at harvest over 3 years (2017–2019) in 17 *Vitis vinifera* L. varietals including the reference cultivar Ortrugo.

Varietals	TSS (°Brix)	pH	TA (g/L)	Tartrate (g/L)	Malate (g/L)	Tartrate-to- malate ratio
Barbesino	21.2^1^ bc	3.03 d	9.09 b	7.69 abc	2.51 cde	4.1 bcd
Bervedino	16.9 fg	3.03 d	6.96 *cd*	5.18 e	1.84 cde	3.4b cd
Besgano Bianco	15.3 *g*	2.80 f	13.43 a	7.32 bcd	7.02 a	1.1 d
Bianchetta di Bacedasco	18.2 ef	3.25 ab	5.49 de	5.98 cde	1.55 de	4.2 bcd
Bianchetta di Diolo	15.4 *g*	3.04 d	9.51 b	5.81 de	4.01 b	1.6 *cd*
Bucalò	25.3 a	3.12 *cd*	6.93 *cd*	7.67 abc	1.66 de	5.0 b
Calora	18.5 e	3.13 *cd*	7.25 c	7.06 bcd	2.06 cde	3.9 bcd
Colombina	20.7 *cd*	2.99 de	6.86 *cd*	5.40 e	2.21 cde	2.6 bcd
Lecco	22.9 b	3.23 bc	6.54 *cd*	7.64 abc	1.97 cde	4.3 bcd
Lisöra	19.1 e	3.05 d	7.99 c	8.08 ab	2.25 cde	3.6 bcd
Marsanne	22.6 bc	3.34 a	4.64 e	5.92 de	1.51 e	4.7 bc
Melara	22.8 b	3.13 *cd*	6.38 *cd*	7.43 bcd	1.83 cde	5.3 b
Molinelli	22.2 bc	3.04 d	9.45 b	8.94 a	2.73 *cd*	3.5 bcd
Ortrugo	20.8 *cd*	3.10 d	5.15 e	6.18 cde	0.46 f	18.3 a
Santa Maria	21.4 bc	3.28 ab	6.83 *cd*	5.95 de	2.96 c	2.4 bcd
Stciucaera Bianca	19.4 de	3.07 d	7.56 c	6.61 bcde	2.42 cde	3.1 bcd
Verdea	15.6 *g*	2.91 e	10.35 b	6.13 cde	4.33 b	1.5 *cd*
**Year**						
2017	21.4 a	3.12 a	6.73 c	6.52 b	1.93 b	5.2 a
2018	18.8 b	3.03 b	7.97 b	7.69 a	2.80 a	5.0 a
2019	19.3 b	3.11 a	8.47 a	6.16 b	2.99 a	2.9 b
**Varietal**	***	***	***	***	***	***
**Year**	***	***	***	***	***	***
**V** × **Y**	ns	ns	***	***	**	**

Data were taken on four vines per varietal. TTS = total soluble solids. TA = titratable acidity. Within each column and factor (varietals and year) mean separation was performed using the Student-Newman-Keuls test. Probability levels are: ***P < 0.001; **P < 0.01; *P < 0.05. ns implies non-significance.

**FIGURE 4:**
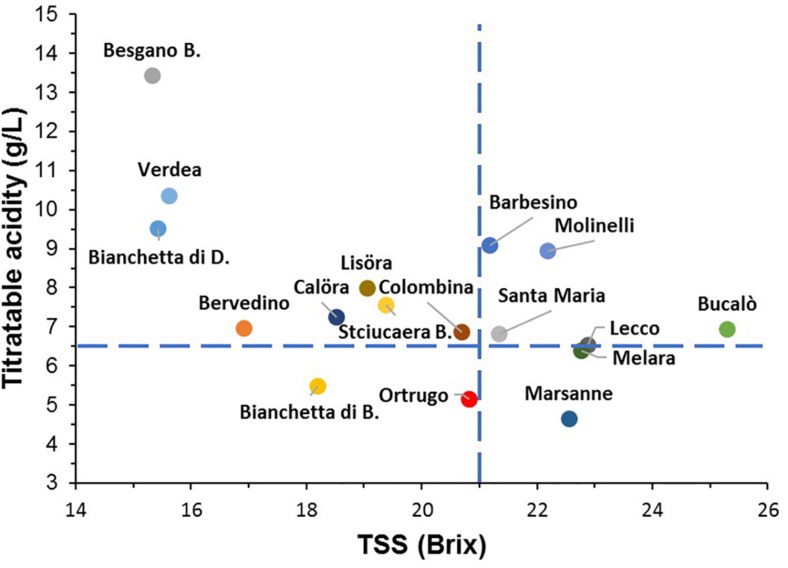
Positioning of the 17 *Vitis vinifera* L. varietals including the reference cultivar Ortrugo as TSS/TA pair data recorded at harvest (data pooled over years). The blue dashed lines indicate requirement thresholds set at a TSS concentration of 21°Brix and a titratable acidity of 6.5 g/L.

When combinations of TSS and TA data were plotted together for all varietals and for data pooled over the 3 years (Figure 4), the reference Ortrugo along with Bianchetta di B. fell inside the bottom-left quadrant showing fairly adequate TSS and too low TA; six other genotypes (Barbesino, Molinelli, Santa Maria, Lecco, Bucalò, and Melara) were grouped within the top-right quadrant identifying requirements for optimal technological maturity in sparkling vinification (TSS ∼ 20–21°Brix and TA ≥ 6.5 g/L). For this reason, these six varietals and the reference Ortrugo were chosen to track seasonal variation of berry mass, TSS, TA, tartrate and malate (pH curves not shown) and were also subjected to Principal Component Analyses.

**FIGURE 5:**
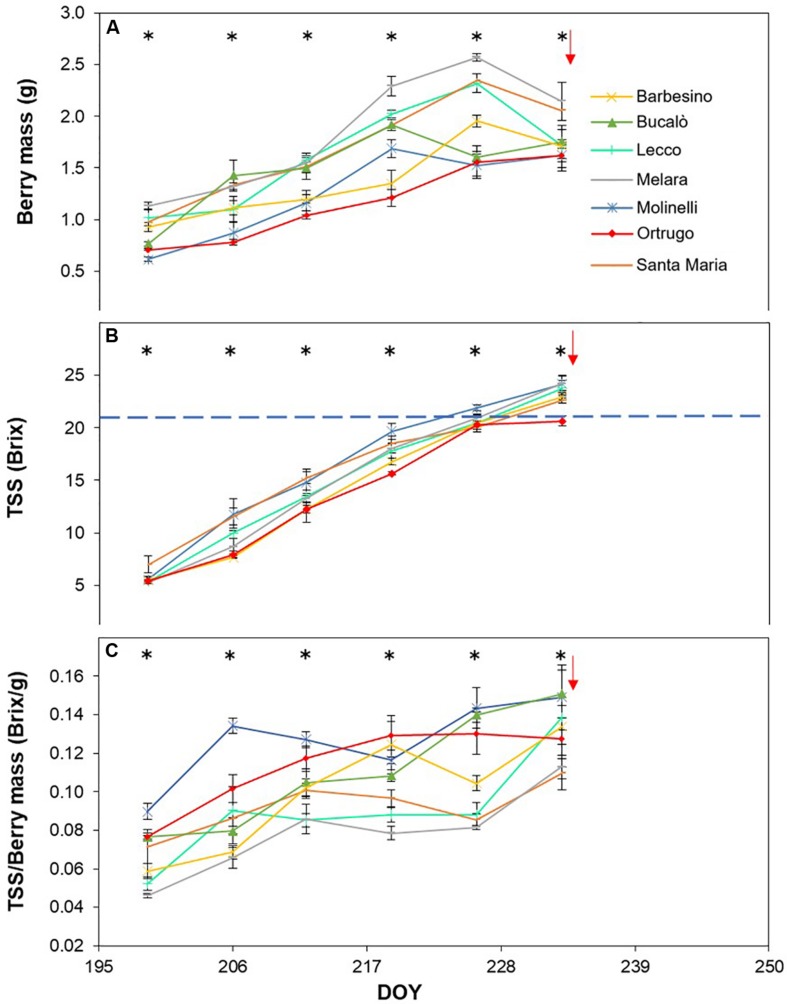
Seasonal evolution of berry mass **(A)**, total soluble solids (TSS) **(B)** and TSS/berry mass ratio **(C)** in 2017 for 7 selected *Vitis vinifera* L. varietals including the reference cultivar Ortrugo. Each point represents the average of three replicates ± SE. Asterisks indicate within-date significant difference at *p* ≤ 0.05. The blue dashed line indicates a TSS concentration of 21° Brix. Red arrows represent the date of harvest. DOY = Day of Year.

**FIGURE 6:**
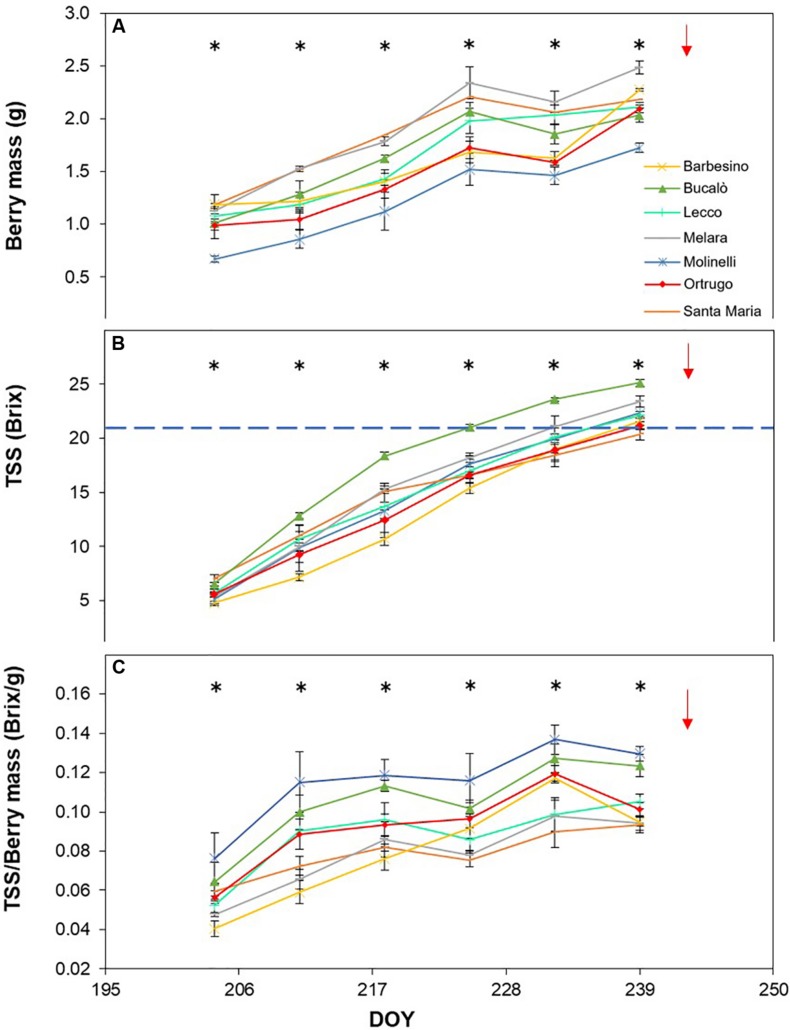
Seasonal evolution of berry mass **(A)**, total soluble solids (TSS) **(B)** and TSS/berry mass ratio **(C)** in 2018 for 7 selected *Vitis vinifera* L. varietals including the reference cultivar Ortrugo. Each point represents the average of three replicates ± SE. Asterisks indicate within-date significant difference at *p* ≤ 0.05. The blue dashed line indicates a TSS concentration of 21° Brix. Red arrows represent the date of harvest. DOY = Day of Year.

**FIGURE 7:**
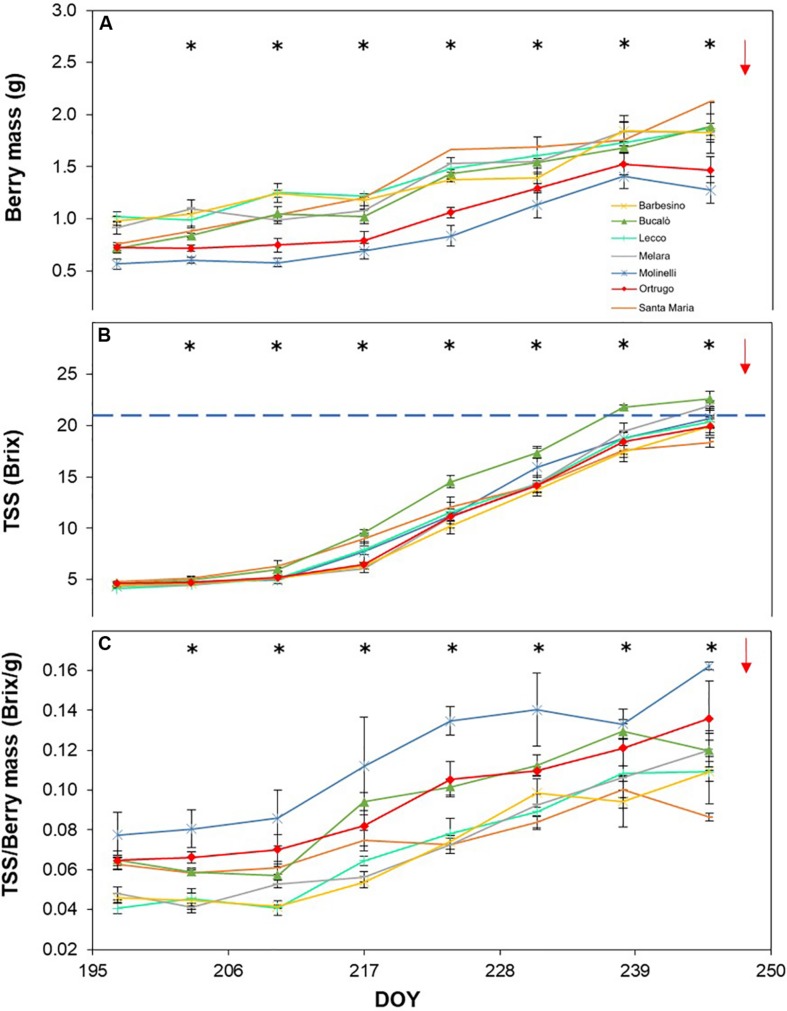
Seasonal evolution of berry mass **(A)**, total soluble solids (TSS) **(B)** and TSS/berry mass ratio **(C)** in 2019 for 7 selected *Vitis vinifera* L. varietals including the reference cultivar Ortrugo. Each point represents the average of three replicates ± SE. Asterisks indicate within-date significant difference at *p* ≤ 0.05. The blue dashed line indicates a TSS concentration of 21° Brix. Red arrows represent the date of harvest. DOY = Day of Year.

Post-veraison seasonal change in berry size showed, in any season, a significant varietal × time interaction and, within each date, significant differences among genotypes (Figures 5A, 6A, 7A). Variation in fraction of berry size formed at the initial measurement over final berry size was mild for data pooled over years (46.7% in 2019 to 48.9% in 2017), whereas the same parameter showed larger variability across varietals ranging from a minimum of 40.7% in Molinelli to a maximum of 55.5% in Lecco. A common feature to every year was that at the very first sampling date large differences in berry size among varietals already occurred; in 2017 such differences tended to be smoothed over time and, at harvest, berry size of the pair Melara and Santa Maria was bigger than the other five varietals all grouped together. Conversely, in 2018 and 2019, initial variation in berry size among genotypes was overall maintained, in relative terms, until harvest.

Total soluble concentration tracked from veraison until harvest shared with berry mass significant varietal × time interaction and, within each date, significant differences among varietals (Figures 5B, 6B, 7B). Notably, in any season, TSS already differed among cultivars at the very first sampling date with Santa Maria invariably having the highest sugar concentration. Such a pattern was quite drastically modified during the ripening phase as, regardless of season, Bucalò always reached maximum TSS at harvest (26.5, 25.1, and 22.6°Brix in 2017, 2018, and 2019, respectively), whereas the lowest final sugar concentration were scored by Ortrugo in 2017 (20.7°Brix), Melara in 2018 (20.4°Brix) and Santa Maria in 2019 (18.4°Brix). Rate of TSS/day increments calculated from first date of sampling until harvest, showed that Bucalò always reached peak values, whereas the lowest rates were measured for Santa Maria and Ortrugo. When data were analyzed as TSS/berry, initial and end-of season values of the different varietals essentially mirrored berry weight trends (Figures 5C, 6C, 7C). In any season, Melara was the most efficient as total sugar accumulated per berry, whereas Ortrugo and Molinelli were the least.

**FIGURE 8:**
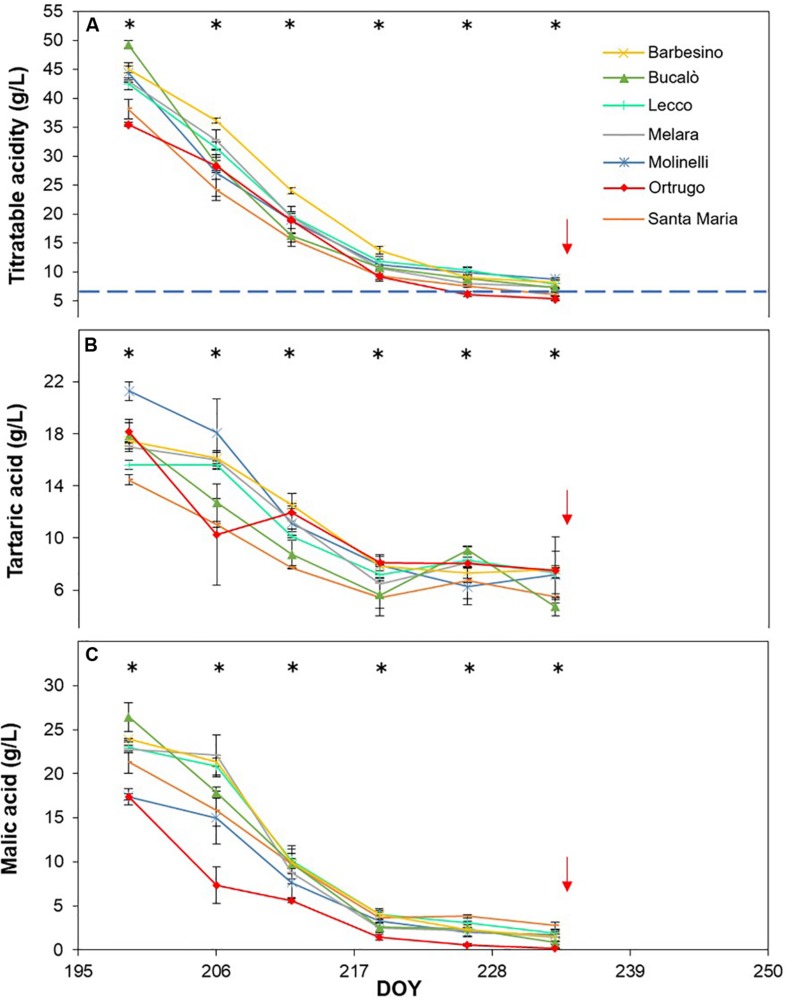
Seasonal evolution of titratable acidity **(A)**, tartaric acid **(B)** and malic acid **(C)** in 2017 for 7 selected *Vitis vinifera* L. varietals including the reference cultivar Ortrugo. Each point represents the average of three replicates ± SE. Asterisks indicate within-date significant difference at *p* ≤ 0.05. The blue dashed line indicates a titratable acidity of 6.5 g/L. Red arrows represent the date of harvest. DOY = Day of Year.

**FIGURE 9:**
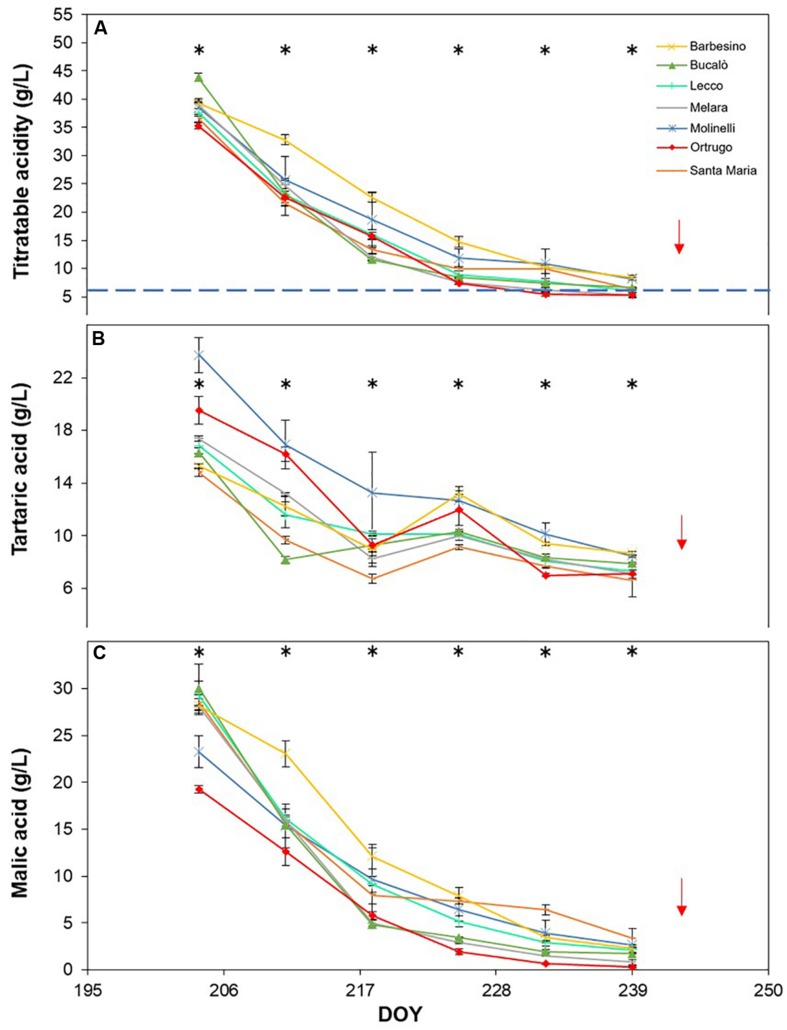
Seasonal evolution of titratable acidity **(A)**, tartaric acid **(B)** and malic acid **(C)** in 2018 for 7 selected *Vitis vinifera* L. varietals including the reference cultivar Ortrugo. Each point represents the average of three replicates ± SE. Asterisks indicate within-date significant difference at *p* ≤ 0.05. The blue dashed line indicates a titratable acidity of 6.5 g/L. Red arrows represent the date of harvest. DOY = Day of Year.

**FIGURE 10:**
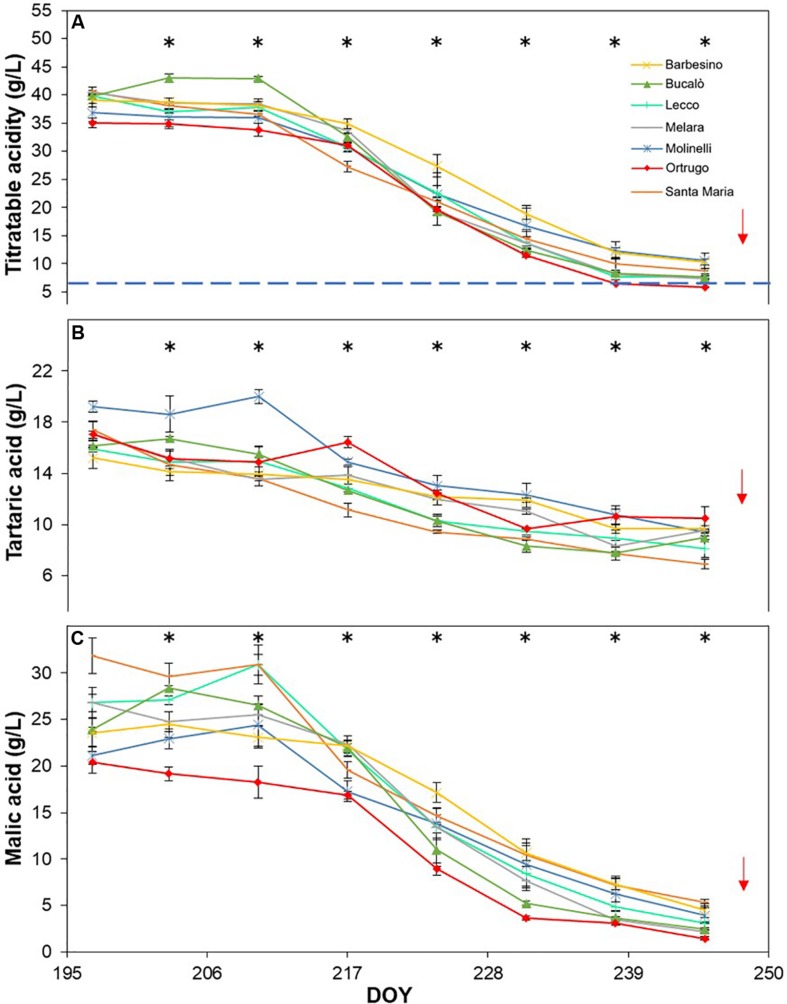
Seasonal evolution of titratable acidity **(A)**, tartaric acid **(B)** and malic acid **(C)** in 2019 for 7 selected *Vitis vinifera* L. varietals, including the reference cultivar Ortrugo. Each point represents the average of three replicates ± SE. Asterisks indicate within-date significant difference at *p* ≤ 0.05. The blue dashed line indicates a titratable acidity of 6.5 g/L. Red arrows represent the date of harvest. DOY = Day of Year.

**FIGURE 11:**
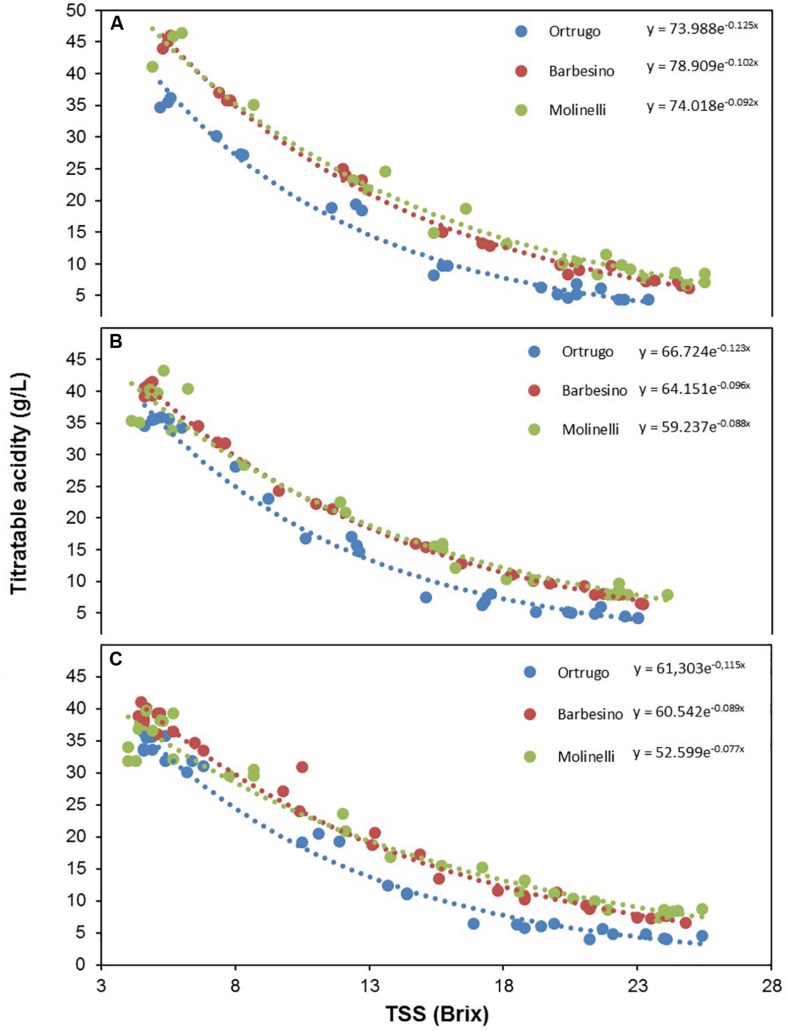
Seasonal variation of titratable acidity expressed as a function of total soluble solids (TSS) in 2017 **(A)**, 2018 **(B)**, and 2019 **(C)**, in Ortrugo (blue), Barbesino (red) and Molinelli (green) grapes. Data were fit to the following equations: Ortrugo 2017 *y* = 73.99e^–0.12x^ *R*^2^ = 0.97; Ortrugo 2018 *y* = 66.72e^–0.12x^ *R*^2^ = 0.98; Ortrugo 2019 *y* = 61.30e^–0.11x^ *R*^2^ = 0.98; Barbesino 2017 *y* = 78.91e^–0.10x^ *R*^2^ = 0.99; Barbesino 2018 *y* = 64.15e^–0.10x^ *R*^2^ = 0.99; Barbesino 2019 *y* = 60.54e^–0.09x^ *R*^2^ = 0.99; Molinelli 2017 *y* = 74.02e^–0.09x^ *R*^2^ = 0.97; Molinelli 2018 *y* = 59.24e^–0.09x^ *R*^2^ = 0.98; Molinelli 2019 *y* = 52.60e^–0.08x^ *R*^2^ = 0.98.

Titratable acidity (TA) measured at first sampling around veraison showed already, every year, a large gap among varietals (Figures 8A, 9A, 10A). Across years, quite consistently, Bucalò had the highest TA values at the beginning of ripening, whereas Santa Maria and Ortrugo had the lowest acid pool. Barbesino and Molinelli were, by far, the two varietals that, regardless of the initial TA level, were able to maintain highest acidity at harvest. When evaluation was made in terms of maintenance, at harvest, of the minimum required TA concentration (6.5 g/L), Ortrugo was never able to meet the requirement. Rate of TA degradation, calculated as TA/day decrease from first sampling date until harvest for each season, was quite accelerated in 2017 (about 1 g/L^∗^day) versus the ∼0.6 g/L^∗^day in the cooler 2019. Notably, Bucalò exhibited the fastest TA degradation rates and Barbesino the lowest, with Ortrugo setting at intermediate positions. When seasonal TSS and TA were plotted together with data pooled over years (Figure 11), an exponential model was fit showing that, over the entire range of represented TSS (about 5 to 25°Brix), Barbesino and Molinelli could account for about 3–5 g/L higher TA than values measured in the reference cultivar Ortrugo.

Seasonal tartaric acid variation differed quite substantially from the TA patterns (Figures 8B, 9B, 10B). The genotype Molinelli was the one showing, upon first sampling, maximum tartrate concentration (19–24 g/L) regardless of season. The same Molinelli along with Barbesino were the two varietals preserving maximum tartrate at harvest (Figures 8B, 9B, 10B and Table 2). Ortrugo showed overall high amounts of tartrate at the beginning and end of the seasonal sampling, scoring maximum pre-harvest concentration in 2019. Rate of tartrate degradation, in the fairly warm 2017 and 2018 seasons, was the fastest in Molinelli and Ortrugo, whereas in 2019 Molinelli and Bucalò marked the most accelerated depletion.

Malate concentration followed from veraison to pre-harvest showed that, at veraison, Bucalò started with the largest pool in 2017 and 2018 (26.4 and 30.0 g/L, respectively), whereas maximum malate concentration was measured in Santa Maria in 2019 (Figures 8C, 9C, 10C). Conversely, every season, Molinelli and Ortrugo started with the lowest malate pools; yet, the fate of those initial amounts was quite different; while Ortrugo retained every year very low amounts (0.46 g/L on a 3 year basis, Table 2), Molinelli showed, even in the hot 2017, a very pronounced ability to preserve malic acid at harvest (2.73 g/L). Though, when the rate of malic degradation was evaluated as malate decrease/day from first date of sampling and harvest, Ortrugo showed quite higher resilience scoring the slowest rate in 2018 (0.496 g/L^∗^day in 2018) and the second slowest rate in 2017 and 2019 after Santa Maria and Molinelli, respectively.

### Principal Component Analysis

**FIGURE 12:**
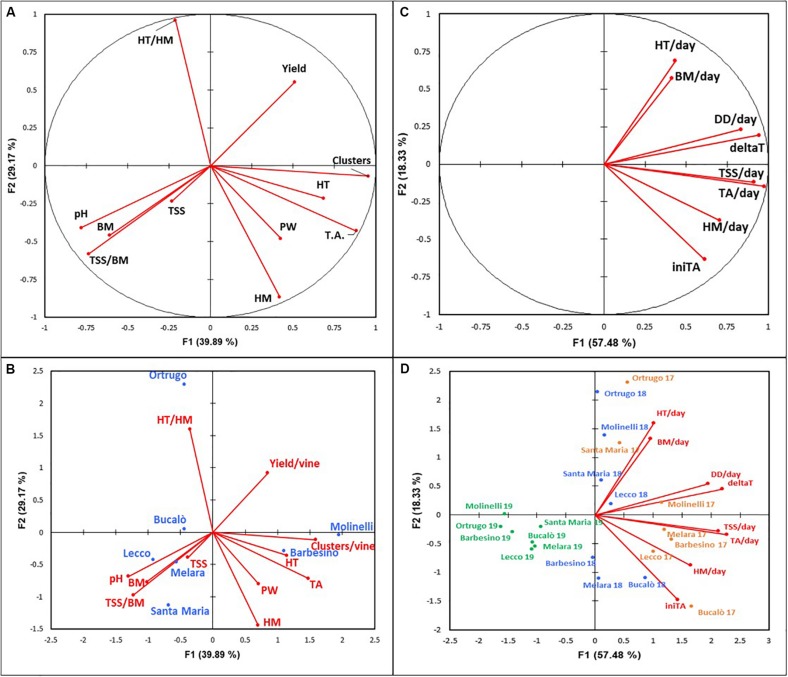
**(A,B)** Principal component analysis (PCA) of 11 variables (axes F1 and F2: 69.06%) for 7 selected *Vitis vinifera* L. varietals, including the reference cultivar Ortrugo (data pooled over 3 years). Panel A represents correlation circle. Panel B represents the biplot with variables and varieties. HT/HM, tartaric acid/malic acid ratio; HT, tartaric acid; TA, titratable acicity; PW, pruning weight; HM, malic acid; TSS, total soluble solids; TSS/BM; total soluble solids/berry mass ratio; BM, berry mass. **(C,D)** Principal component analysis (PCA) of 8 variables (axes F1 and F2: 75.81%) for 7 selected *Vitis vinifera* L. varietals, including the reference cultivar Ortrugo (data pooled over 3 years). Panel C represents the correlation circle. Panel D represents the biplot with variables and varieties. Varietals are reported according to their bahaviour in the 3 years of trial (2017 in orange, 2018 in blue, 2019 in green). HT/day, tartaric acid per day; BM/day, berry mass per day; DD/day, degree day per day; deltaT, (Tmax – Tmin)/day; TSS/day, total soluble solids per day; TA/day, titratable acidity per day; HM/day, malic acid per day; iniTa, initial titratable acidity.

Principal component analysis was run first on data pooled over years for the seven selected varietals and for 11 variables representative of vegetative growth, yield and grape composition. PCA F1 and F2 dimensions were those covering the highest variability (69.06%) against 63.12% covered by F1 and F3 and only 52.40% explained by F2 and F3. Therefore, F1 and F2 dimensions were chosen to plot the correlation circle (Figure 12A) and the observation bi-plot (Figure 12B).

Looking at different reciprocal angles formed by the direction of the different PCA vectors, it was apparent that TSS (Brix) held a negative relationship with yield and pruning weight per vine (*r* = −0.675 and −0.605, respectively, and the same negative linear model was found for tartrate vs. berry weight (*r* = −0.668). Interestingly, the same significant relationship was not shown when berry weight was regressed against malate (*r* = 0.192). A positive correlation was found for TA vs cluster number (*r* = 0.833) and for malic acid and pruning weight (*r* = 0.663).

The observation bi-plot allowed a quite clear separation of different varietals. The reference Ortrugo is isolated from the remaining varietals due to an inverse correlation between tartrate/malate ratio (very high) and malate (very low) and a good attitude to crop under a fairly low vigor status. The bottom-right quadrant of the observation bi-plot also isolates cvs. Molinelli and Barbesino, these sharing a distinct attitude of maintaining high TA primarily through high tartrate retention. Yield and sugar accumulation are sufficient whereas vine vigor is higher than Ortrugo. Then a third group, albeit more dispersed, encompasses Bucalò, Melara, Santa Maria and Lecco which are reunited by the capacity to achieve high sugar concentration and content, high pH and berry size. Their crop load is generally moderate or low.

A second PCA run was performed on the same seven varietals using yearly data of TSS and acid components rates as well as of DD/day and (T_max_ – T_min_)/day within the time period comprised between first grape sampling date and harvest. This latter corresponded to 35, 38, and 51 days in 2017, 2018, and 2019, respectively.

The correlation circle (Figure 12C) showed some expected positive correlations (e.g. those between DD/day and (T_max_-T_min_)/day vs. daily rates of TSS increment and TA degradation). A good correlation (*r* = 0.704) was also found between DD/day and rate of malate degradation, whereas tartrate degradation was less responsive to active temperatures. Interestingly, no correlation was found between daily degradation rates of malic and tartaric acids.

The observation bi-plot which maintained single season behavior of different varietals was quite effective at separating 2019 behavior versus the 2017 and 2018 patterns with varietals that tended to mix. Though, in both 2017 and 2018, Ortrugo and Molinelli consistently separated due to their characteristic to show faster rates of diminishing tartaric acid concentration; by contrast Melara, Lecco and Bucalò grouped together for their high rates of malate depletion. Then, Barbesino and Santa Maria positioned themselves according to year; Barbesino in 2018 showed strong retention capacity for tartaric acid, whereas Santa Maria in 2017 had quite high rates of tartaric acid decrease.

## Discussion

Criteria chosen in our work to establish minimum desirable requirements for sparkling wine making (TSS around 20–21°Brix and TA ≥ 6.5 g/L) were quite effective to narrow down to 6 varietals, from an initial batch of 16, the group size within which reliable alternatives to the reference Ortrugo could have been found. Data shown in Tables 1, 2 as well as PCA run on 11 variables representative of growth, yield and grape composition (Figure 12B) have clearly isolated the behavior of Ortrugo vs. the remaining varieties, confirming its weaknesses at maintaining, regardless of the seasonal weather, enough acidity to allow proper sparkling wine making. Such inadequacy manifests through a very high tartrate-to-malate ratio, which, in turn, originates from extremely low malic acid retention at harvest. However, final TA recorded at harvest is the result of complex interactions among factors affecting synthesis, dilution and degradation of different acids throughout the season.

Pre-veraison tartaric acid pool available in Ortrugo berries seems adequate as the measured 18.2 g/L (average over 3 years) ranks second after the top scorer Molinelli (21.4 g/L). It has been well established in literature (Walker and Famiani, 2018) that ascorbic acid must be considered the true intermediate precursor in grape tartrate biogenesis. This was resolved in an experiment where young leaves were fed with ^14^CO_2_ or [U-14C]-sucrose and the accumulation of radiolabel could be followed over time into glucose/ascorbate-2-keto-L-idonate/L-idonate/5-keto-D-gluconate and finally tartaric acid (Hawker, 1969). Moreover, while it has been ascertained that the immature berry itself is the main, if not unique, site of tartaric acid biosynthesis (Hale, 1962) it has been shown in a molecular study that levels of transcripts encoding L-IdnDH were down-regulated in berries grown in the dark using clusters inserted in light-excluding boxes (De Bolt et al., 2008). Such scientific evidence leads to conclude that abundance of glucose substrate as well as an open canopy allowing good cluster exposure to light might be beneficial for accumulation of tartaric acid in the green berry. As a matter of fact, Ortrugo seems to satisfy both requirements; on a 3 year basis, it is the varietal showing the highest amount of total sugar per vine (493 g/vine) and both Ravaz index (8.6) and total pruning weight per vine (308 g/m of row length) are quite typical of low vigor conditions, ruling out the possibility of excessive cluster shading by a too dense canopy. According to Ruffner (1982), tartaric acid increases in the green berry until about 4 weeks after flowering. Thereafter, its synthesis is halted and further changes in concentration are almost exclusively due to increasing berry volume during the post lag growth phase, leading to dilution of tartaric acid (Ford, 2012). Thermal stability is also common and shared knowledge of physiological biochemistry of tartaric acid whose concentration in the berry is essentially unaffected by temperature; some pioneer work alluding to a “respiration” of tartaric acid at temperatures above 30°C (Peynaud and Maurié, 1958) never found subsequent validation. Further, if dilution is the main player in the dynamic of tartaric acid decrease after veraison, observation-bi plot shown in Figure 12D clearly indicates that daily increment in berry fresh mass (BM/day) and daily decrease in tartaric acid concentration (HT/day) were correlated and such correlation was mostly reflected by 2017 and 2018 Ortrugo data. Overall, in term of tartaric acid seasonal balance, main weakness for Ortrugo was quite high dilution rates rather than a limitation in the initial pre-veraison build up.

As for malic acid, Ortrugo started each season with the lowest amounts as compared to any other varietals; initial malic concentration in green berries measured in Ortrugo was 19.0 g/L (data pooled over years) with a maximum gap vs. Bucalò (starting at 26.8 g/L). Malate biosynthesis starts in the immature berries about 7 days after flowering whereas the onset of softening marks the beginning of a rapid loss (Coombe, 1995; Ollat et al., 2002). Hale (1962) demonstrated that malate is synthesized in the berry and not imported from any other part of the vine. There is also a shared consensus that the primary pathway for malic acid formation in the green berry is through the cytoplasmic enzyme phospho-enol-piruvate (PEP) carboxylase which catalyzes the B-carboxilation of PEP arising from glycolysis, forming oxaolacetic acid; such reaction is often referred as “dark fixation of CO_2_” (O’Leary, 1982). Indeed, several other enzymes have been associated with malate breakdown, including those associated with gluconeogenesis, respiration and fermentation (Ruffner, 1982; Famiani et al., 2016). As related to the effect of environmental factors on malate accumulation, although no evidence has been reported in terms of sensitivity to radiation, Buttrose et al. (1971) have confirmed that high temperature during the pre-veraison period led to lower levels of malate accumulation. According to the above scenario, reasons explaining why Ortrugo shows a scarcer malate pool at pre-veraison, if compared to other varietals, still remain in the realm of speculation. Indeed, taking the time window between 1 June and 15 July within which the first stage of berry growth and lag phase can be reasonably placed, it seems true that the cooler 2018 (average daily T_max_ = 27.9°C and DD = 567) did result in higher malate accumulation in all varietals except for Ortrugo, therefore showing its quite low sensitivity to such thermal trigger. Then, if it is true that rates of dark (night) respiration are also linked to net photosynthesis rates during the preceding day (Poni et al., 2006), canopies having high carbon balance might be favored at accumulating malate. However, in this study we neither recorded leaf gas exchange parameters nor did we estimate canopy light interception which, to a certain extent, is a good estimator of total canopy photosynthesis (Poni et al., 2003). The closer approximation in our trial is given by the Ravaz index, values of which ranging between 5 and 6 are supposed to correspond to adequate vine balance (Howell, 2001); among the seven selected varietals, though, Ortrugo is the one having the highest ratio (8.60) suggesting a vine balance leading to a quite limited canopy supply function as compared to sink demand.

Actually, besides the low malate pool at veraison, Ortrugo has also shown a very scarce ability in preserving a sufficient malic acid share from degradation. Over 3 years, the concentration at the end of the season was just 2.5% of that found at first samplings, whereas other genotypes such as Barbesino and Molinelli maintained higher fractions (7.6 and 8.8%, respectively, of the concentration at veraison). Post-veraison malate degradation is enhanced by high temperatures (Buttrose et al., 1971), whereas an active role of the light regime has never been demonstrated in contrast to the effects seen on tartaric acid and ascorbate (Dokoozlian and Kliewer, 1996). As a confirmation, PCA reported in Figures 12C,D shows no correlation between daily loss of malic and tartaric acid throughout the post-verasion period until harvest. However, Ortrugo does not have any faster consumption of the initial malic acid pool since rates of malic degradation were, on a 3 year basis, the lowest (0.45 g/L^∗^day) among the seven varieties. Although Ortrugo’s weakness at retaining adequate TA at harvest has already been reported in some specific papers (Gatti et al., 2015, 2019), this study clarifies that such limitation primarily rises from fairly low malic acid accumulation pre-veraison and intense dilution of tartaric acid after veraison. Figure 11, showing the decreasing trend of TA in Ortrugo, Molinelli and Barbesino vs increasing TSS, is quite self-explanatory: the three patterns do not differ in terms of slopes rather for a given intercept values that stays quite constant throughout the whole ripening season.

Looking for alternative genotypes ensuring the economic sustainability of the productive process, a satisfying yield is still a basic requirement (Howell, 2001). In our work, most of the minor local genotypes from the Colli Piacentini area stood out for an average productivity comparable to Ortrugo, the main varietal elected for the local appellation (Table 1). Considering that Ortrugo, in the most favorable vintages, sets very close to the maximum yield allowed of 12 tons/ha, all those minor cultivars exhibiting yields similar to Ortrugo can be considered performant genotypes in terms of average productivity. In particular, the good basal nodes fruitfulness demonstrated by Bervedino, Molinelli and Lisöra (Table 1), make these cultivars very prone to a modern vineyard management based on spur-pruning and mechanization of pre-pruning operations (Poni et al., 2016).

PCA analyses shown in Figure 12B isolate within the bottom-right quadrant the behavior of Molinelli and Barbesino. Molinelli stands out as an ideal cultivar for sparkling wine purposes as it exhibits desirable agronomic features (such as balanced cropload, small and loose clusters, fairly high fruitfulness of the basal nodes), fast sugar accumulation and a sort of extraordinary ability to retain TA with a well-balanced tartrate-to-malate ratio. Notably, Molinelli retained enough malic acid (about 2 g/L) even in the hot 2017 not because its daily degradation rate was slower than Ortrugo, rather because the initial pool was higher. Similar performances were also shown by Barbesino, which, however, showed more sensitivity than Molinelli to malic acid degradation. Current appellation regulation for Ortrugo allows a maximum 10% of “other varieties” to be blended and the mix with Molinelli or Barbesino, once registered, seems highly recommended.

Finally, PCA analyses allowed the grouping of the four remaining varietals (Bucalò, Lecco, Santa Maria and Melara) showing a generally high rate of sugar accumulation and maintenance of adequate TA. However, possible use of such genotypes seems to be spoiled by low productivity that, especially in Lecco, Bucalò and Melara, diminishes the interest as alternate choices for Ortrugo.

## Conclusion

A detailed 3 year study on agronomic performance of 16 minor white varietals of the Colli Piacentini grape district was performed to assess if any of them could provide a reliable alternative to the local main white cultivar Ortrugo, showing major limitations at maintaining sustained acidity at harvest in the context of global warming. In regard to the mechanisms leading Ortrugo to present, at harvest, low TA with just traces of malic acid, it has been ascertained that the two most limiting factors are post veraison intense dilution of tartaric acid and too low malic acid accumulated pre-veraison.

Data processing assisted with both repeated measures and principal components analyses allowed a thinning down of the initial group of 16 genotypes to 7 varietals (Molinelli, Barbesino, Bucalò, Melara, Lecco, Santa Maria and the reference Ortrugo) showing ability to combine at harvest adequate TSS (20–21°Brix) and minimum required TA (≥6.5 g/L). Then, further analyses have shown that especially Molinelli and Barbesino, regardless of season, performed very efficiently as optimal technological maturity for sparkling wine making, productivity, vine balance and cluster morphology. Such two minor varietals stand out as ideal alternatives to Ortrugo and could contribute to the enhancement of the industry competitiveness by posing new products with an unaffected link to the district identity.

Our work demonstrates that grapevine intra-specific biodiversity hides prominent potentialities for viticulture adaptation strategies to climate change and renew the emphasis on the value of genetic resources conservation.

## Data Availability Statement

The raw data supporting the conclusions of this article will be made available by the authors, without undue reservation, to any qualified researcher.

## Author Contributions

TF, MG, and SP designed and supervised the research. TF, GB, AG, CS, LR, and MG performed the research and analyzed the data. TF, GB, MG, and SP drafted the manuscript. CS, AG, and LR critically revised the manuscript. LR verified quality of written English. All authors read and approved the final manuscript.

## Conflict of Interest

The authors declare that the research was conducted in the absence of any commercial or financial relationships that could be construed as a potential conflict of interest.
