# Supplementary material for: Biodiversity of Local Vitis vinifera L. Germplasm: A Powerful Tool Toward Adaptation to Global Warming and Desired Grape Composition
Source: Front Plant Sci. 2020 May 14;11:608. doi: 10.3389/fpls.2020.00608 (PMC7240219; doi:10.3389/fpls.2020.00608)
Supplement: Supplementary file 1 [file Data_Sheet_1.docx]

Supplementary Material

# Supplementary Figures

Figure S1. Representative clusters of 16 minor white grapes varietals and of the reference cultivar Ortrugo compared over 2017, 2018, 2919 for suitability to sparkling wine making.

Figure S2. Seasonal daily trends of minimum temperature (red lines), mean temperature (violet lines), maximum temperature (green lines) and rainfall (blue bars) in 2017 (A), 2018 (B), and 2019 (C). Red arrows indicate the date of harvest. DOY= Day of the Year.

# Supplementary tables

Table S1. Rainfall and heat accumulation (Growing Degree Days, GDD) recorded at the experiment site in 2017, 2018 and 2019.

| Year | Harvest date | Harvest DOY^1^ | Rainfall  1 Apr - 31 Oct | GDD  1 Apr -31 Oct | GDD  1 Apr - 30 Sep |
| --- | --- | --- | --- | --- | --- |
|  |  |  | (mm) | (GDD) | (GDD) |
| 2017 | 22 August | 235 | 289 | 2143 | 1984 |
| 2018 | 30 August | 243 | 433 | 2200 | 2014 |
| 2019 | 5 September | 249 | 595 | 2020 | 1963 |

^1^ DOY = Day of the year
